# Medical News and Items

**Published:** 1878-05

**Authors:** 


### Medical News and Items.

American Medical Association.—The twenty-ninth annual session will be held in the city of Buffalo, N. Y., on Tuesday, Wednesday, Thursday, and Friday, June 4, 5, 6, and 7, commencing on Tuesday at 11 a. m.

" The delegates shall receive their appointment from permanently organized State medical societies, and such county and district medical societies as are recognized by *representation in their respective State societies*, and from the medical department of the army and navy of the United States."

" Each State, county and district medical society entitled to representation, shall have the privilege of sending to the association one delegate for every ten of its regular resident members, and one for every additional fraction of more than half that number: *Provided*, however, that the number of delegates for any particular State, territory, county, city or town shall not exceed the ratio of one in ten of the resident physicians who may have signed the code of ethics of the association."

Sections.—Practice of medicine, materia medica and physiology: Dr. A. L. Loomis, New York, Chairman; Dr. J. H. Etheridge, Chicago, Ill., secretary. Committee appointed to report to this section: On clinical and meteorological records: Dr. N. S. Davis, Illinois, chairman. Obstetrics and diseases of women and children; Dr. E. W. Jenks, Detroit, Mich., chairman; Dr. H. O. Marcy, Cambridge, Mass., Secretary. Surgery and anatomy: Dr. Henry H. Smith, Philadelphia, Pa., chairman; Dr. E. T. Easley, Little Rock, Ark., secretary. Medical jurisprudence, chemistry and psychology; Dr. Walter Kempster, Oshkosh, Wis., chairman; Dr. E. A. Hildreth, Wheeling, W. Va., Secretary;

State Medicine and Public Hygiene: Dr. J. L. Cabell, University of Va., chairman; Dr. E. J. Marsh, Paterson, N. J., secretary.

The following committees are expected to report: On prize essays; Dr. E. M. Moore, Buffalo, N. Y., chairman. On necrology; Dr. J. M. Toner, Washington, D. C., chairman. On catalogue of national library; Dr. H. C. Wood, Pa., chairman. On recommendations in President Bowditch's address; Dr. N. S. Davis, Illinois, chairman.

The following papers are to be presented at the meeting of the surgical section: Address by Henry H. Smith, M. D., chairman of the section, on certain points in the pathology of the bones, including tubercles. On disease germs, their nature, origin, and relations in cases of wounds, by B. A. Watson, M. D., Jersey City. On Septicæmia after re-sections, by D. H. Weeks, M. D., Portland, Me. On tracheotomy without tubes, by Henry A. Martin, M. D., Boston, Mass. On identity of hospital gangrene with diphtheria, by John T. Carpenter, M. D., Pottsville, Pa. On permeability of entire alimentary canal by enemata with some surgical applications, by Robert Battey, M. D., Rome, Georgia. On irritation of the metatarso-phalangeal articulation in valgus of the great toe, by Frank H. Hamilton, M. D., New York. On the process of repair in wounds with and without antiseptic treatment, by Frederic Hyde, M. D., Cortland, N. Y. On extirpation of the thyroid gland, by Julius F. Miner, M. D., Buffalo, N. Y. On fracture at the wrist, by John H. Packard, M. D., Philadelphia, Pa. On pathology and treatment of cancer, by Theodore A. McGraw, M. D., Detroit, Mich. On perityphlitic abcess, by D. M. Clay, M. D., of Shreveport, La.

All papers to be presented in the session of the section should be forwarded to Henry H. Smith, M. D., chairman of the surgical section, No 1800 Spruce street, Philadelphia.

At the annual meeting of the Michigan State Board of Health, which was held at Lansing, Tuesday, April 9, 1878, president Kedzie presented his annual address on "The Work of the State Board of Health," in which he gave an account of the past work of the Board and outlined its work for the immediate future.

Among the many duties which the board had performed since its organization, about the first effort was for the establishment of well organized and effective Boards of Health in every township, city and village throughout the State, and then bringing the State Board of Health into communication and active co-operation with all these local Boards of Health, thus gaining two important objects ; (1) having an effective channel for imparting information ; (2) having organized bodies through which the statistics in regard to public health could be gathered from all parts of the State. Besides this the board had secured the assistance of many physicians throughout the State, receiving from them many valuable reports, communications, and replies to circulars regarding the cause and progress of various diseases. In speaking of the efforts to impart information and gather statistics bearing on the public health he said the results were most gratifying. Not only sanitarians but the people at large, are grasping that very important idea, the possibility of the prevention of disease and death ; that many diseases may be prevented altogether, or that when they do appear they may be stamped out as a forest fire may be extinguished, or they may be walled in like an inundation.

In outlining the future work of the board, the Doctor said that the law now says that the board shall from time to time recommend standard works on hygiene to be used as text-books in our common schools. He recommended that this subject be referred to a committee to report to the board at an early day.

He recommended the early publication of the material collected by the board, such as meteorological data, reports of diseases, etc.

Inasmuch as the methods for disinfection are similar for different diseases, he recommended that a general circular on the use of disinfectants be prepared and issued, so that whenever the board wishes to issue instructions for the prevention of any self-propagating disease, such a circular will save repetitions.

The subject of the pollution of streams by sewage is a question of great importance, and one that should be settled before sewer systems become practically beyond control on account of the large outlay of money to effect a change. When Detroit first emptied its sewage into the river it was claimed that it was not possible to pollute such a mass of water, but since the country has become thickly settled above, the water supply of Detroit is already endangered, and fears are expressed that as the population above Detroit along the river becomes greatly increased the water of Detroit river will become unfit for domestic use.

He suggested the holding of sanitary conventions in different parts of the State to discuss sanitary subjects, and to bring together dealers in sanitary appliances, sanitary experts, and the people generally who need instruction in sanitary work. He hoped to see the day when these questions which lay hold of life shall be as freely discussed by the laity as they now are by experts in sanitary science. If such conventions were held, the secular press would distribute widely the truths brought out.

ILLINOIS STATE MEDICAL SOCIETY.—The Twenty-eighth Annual Session of this Society will be held in the city of Springfield, Ill., on the 21st, 22nd and 23d days of May, 1878. The president of the Association for the current year is Dr. J. L. White, of Bloomington. The Committee of Arrangements is composed of Drs. P. M. Griffith, J. Townsend and A. N. Patterson, all of Springfield. It is expected that there will be an unusually interesting session, as the attendance promises to be larger than heretofore, and many valuable papers are promised.

A NEW MEDICAL JOURNAL.—Beginning with the first of July, 1878, there will be issued from Cincinnati a Monthly Journal, to be known as the *Obstetrical Gazette.* It will contain forty-eight pages monthly, size and style of the *Popular Science Monthly.* It will be devoted exclusively to the cultivation and promotion of knowledge in Obstetrics, Gynecology, and Diseases of Children.

It will be edited by E. B. Stevens, A. M., M. D., for eighteen years editor of the *Cincinnati Lancet and Observer.*

A PHYSICIAN of *stamina* and *experience*, is called for in a thriving Indiana town. Address for particulars, etc., J. H. Etheridge, M. D., 603 Michigan avenue, Chicago, Ill.

The Regular Annual Meeting of the Association of American Medical Editors, will be held on Monday evening, June 3d, 1878, at the Tifft House, Buffalo, N. Y. All Editors of American Medical Journals are eligible for membership, and are cordially invited to be present and participate in the meeting.

